# Effects of Neck Radiation Therapy on Carotid Arteries in Patients with Head and Neck Carcinoma

**DOI:** 10.31661/gmj.v13i.3363

**Published:** 2024-09-09

**Authors:** Hosein Aghamiri, Amin Rahmani, Mir Ramin Khatib Shahidi, Shaghayegh Kamian, Ahmad R Mafi

**Affiliations:** ^1^ Neurology Department, Shahid Beheshti University of Medical Sciences, Imam Hosein Hospital, Tehran, Iran; ^2^ Radiation Oncology Department, Shahid Beheshti University of Medical Sciences, Imam Hosein Hospital, Tehran, Iran

**Keywords:** Radiotherapy, Ultrasound Results, Carotid Arteries, Head and Neck Cancer

## Abstract

Background: Radiotherapy is a main treatment modality for cancers of the head and neck (HNC). However, it can cause a number of complications, including carotid artery damage.This study aimed to determine the effect of head and neck radiotherapy on carotid Doppler ultrasound findings in patients with HNC treated at Imam Hossein University Hospital. Materials and Methods: This research is a descriptive-longitudinal and prospective study conducted in 2022 on patients with HNC undergoing neck radiotherapy. In this study, before the initiation of radiotherapy, the patients underwent neck carotid artery Doppler ultrasound and various parameters including peak systolic velocity (PSV), end-diastolic velocity (EDV), internal carotid artery/common carotid artery (ICA/CCA) PSV, the number of plaques, intimal thickness, and percentage of carotid diameter stenosis, were recorded. The Doppler ultrasound was repeated six months later, addressing the same characteristics, and the collected data were compared. Results: Of 49 investigated patients, 32 (65.3%) were male (mean age=59.46 years). PSV, EDV, and ICA/CCA ratios showed no statistically significant difference 6 months following the completion of radiotherapy compared to the initial investigation. On the other hand, the percentage of carotid artery stenosis, the middle intimal membrane thickness, and the number of vascular plaques increased significantly (P0.05). Conclusion: Carotid artery stenosis due to radiotherapy seems to be a common complication; however, radiotherapy probably has minimal effects on other ultrasound parameters of carotid arteries including EDV, PSV, and ICA/CCA ratio.

## Introduction

Based on recent data, lip, oral cavity, and pharyngeal cancers are responsible for approximately 3.2 percent of global cancer deaths [1]. Head and neck cancers (HNC) are the seventh most common cancer worldwide, encompassing malignancies originating from various areas such as the skin, nasal cavity, oral cavity, pharynx, and larynx. The most prevalent type of HNC is squamous cell carcinoma, with other types including adenocarcinomas, adenoid cystic carcinomas, lymphomas, and plasmacytoma [2][3][4][5]. They account for approximately 4% of malignancies with a 10-50% five-year recurrence rate [6][7] and are responsible for about half a million annual deaths worldwide [8][9][10][11]. HNC treatment typically involves a combination of surgery, radiotherapy, chemotherapy, targeted therapy, and immunotherapy [4][5][8][12][13][14]. Radiotherapy is a key treatment modality for HNCs, although it can lead to complications such as carotid artery stenosis [15][16]. As radiotherapy more often affects the cells with a higher proliferation rate (epithelial, and endothelial cells), these cells are more prone to develop radiation-induced complications [7][17].

Despite improvements in the planning and delivery of radiotherapy, carotid artery stenosis remains one of the notable adverse effects of radiotherapy in HNC survivors [18][19][20]. Previous studies have demonstrated that significant changes can occur in the carotid arteries and other vessels following head and neck radiotherapy [21][22]. Carotid artery Doppler ultrasound is a reliable, sensitive, and non-invasive method for the diagnosis of carotid artery disorders [23][24]. This study aims to explore the impact of neck radiotherapy on carotid artery Doppler ultrasound results in HNC patients.

## Materials and Methods

**Table:** Table1. Demographic Characteristics of Patients Participating in the Study

**Variable**	**Sub-Variable**	**Frequency**	**Percentage**
Gender	Male	32	65.3
	Female	17	34.7
Marital status	Single	6	12.7
	Married	43	87.8
	Under diploma	9	18.4
Educational level	Diploma	12	24.5
	Associate	5	10.1
	Bachelor	19	38.8
	Master and higher	4	8.2
	Employee	11	22.4
	Freelancer	18	36.7
Occupation	Housewife	9	18.4
	Retired	11	22.4
	Poor	8	16.4
Economic status	Moderate	35	71.4
	Good	6	12.2
Place of residence	City	36	73.5
	Village	13	26.5
A family history of cancer	No	37	75.5
	Yes	12	24.5
Alcohol use	No	41	83.7
	Yes	8	16.3
Cigarette use	Yes	33	67.3
	No	16	32.7
	Right	13	26.5
Carotid arteries exposed to radiotherapy	Left	15	30.6
	Both sides	21	42.9

This study is a descriptive-longitudinal prospective study conducted on 49 patients with HNC who were referred to the Department of Clinical Oncology, Imam Hossein Hospital and underwent head and neck radiotherapy in 2022 and 2023. The inclusion criteria included patients with a definite diagnosis of HNC that needed radiotherapy to the neck area, who were willing to participate in the study. The exclusion criteria included patient death for any reason before the second investigation and consent withdrawal. A checklist was used to collect the data including demographic characteristics and ultrasound findings of the patients, including peak systolic velocity (PSV), end-diastolic velocity (EDV), ICA/common carotid artery (ICA/CCA) PSV, the number of plaques, intimal thickness, and percentage of carotid diameter stenosis, assessed before the initiation and 6 months after the completion radiotherapy. According to the of Akhan et al. [21] with assumption of Diameter stenosis of Less than 50% in pre-irradiation and post-irradiation, equal to 8.8% () and 51.1% (), respectively, α=0.01 and β=0.1 and by use of the following sample size formula, the obtained minimum sample size (n) is 28, but due to the fact that we had more samples in this study, 49 people were included in each of the groups under study.

**Figure:**
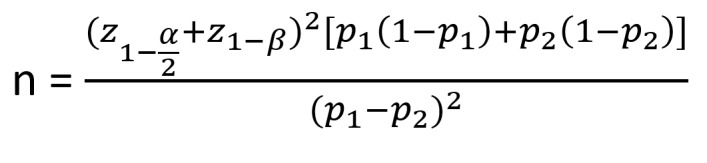


Statistical Method and Data Analysis

The data were analyzed using the Statistical Package for the Social Science-27 (IBM SPSS Statistics for Windows, version XX (IBM Corp., Armonk, N.Y., USA)). The results of quantitative data and qualitative data were reported as mean ± standard deviation and number (percentage), respectively. Paired t-test was used to compare the mean of quantitative variables (the middle intimal membrane thickness and the number of plaques) before and 6 months after radiotherapy. The McNemar test was used to compare the frequency distribution of qualitative variables (PSV, EDV, carotid diameter size, and the classified ICA-to-CCA ratio) before and 6 months after radiotherapy. The normality of the frequency distribution of the quantitative variables was assessed using the non-parametric Kolmogorov-Smirnov test, and the equality of variance of the groups was assessed via Levene’s test. The significance level in the tests was considered 0.05 (Table-2).

## Results

**Table:** Table2. Comparison of Peak Systolic Velocity in Carotid Arteries before and 6 Months after Radiotherapy

**Carotid Type**	**PSV (CM/S)**	**Before Radiotherapy **		**6 Months After Radiotherapy **		
		**Frequency**	**Percentage**	**Frequency**	**Percentage**	**P-value**
	Less than 125	34	100	32	94.1	
Right carotid	125-230	0	0	2	5.9	0.5*
	More than 125	0	0	0	0	
	Invisible	0	0	0	0	
	Less than 125	36	100	35	97.2	
Left carotid	125-230	0	0	1	2.8	1*
	More than 125	0	0	0	0	
	Invisible	0	0	0	0	
	Less than 125	70	100	67	95.7	
Sum of both	125-230	0	0	3	4.3	0.25*
	More than 125	0	0	0	0	
	Invisible	0	0	0	0	

**PSV:** Peak systolic velocity, McNemar Test*^*^

**Table:** Table3. Comparison of End-diastolic Velocity in Carotid Arteries before and 6 Months after Radiotherapy

**Carotid Type**	**EDV (CM/S)**	**Before Radiotherapy **		**6 Months After Radiotherapy**		
		**Frequency**	**Percentage**	**Frequency**	**Percentage**	**P-value**
	Less than 40	34	100	33	97.1	
Right carotid	40-100	0	0	1	2.9	1*
	More than 100	0	0	0	0	
	Less than 40	36	100	33	91.7	
Left carotid	40-100	0	0	3	8.3	0.25*
	More than 100	0	0	0	0	
	Less than 40	70	100	66	94.3	
Sum of both	40-100	0	0	4	5.7	0.125*
	More than 100	0	0	0	0	

**EDV:** End-diastolic velocity, McNemar Test^*^

**Table:** Table4. Comparison of the Percentage of Carotid Artery Stenosis before and 6 Months after Radiotherapy

**Carotid Type**	**percentage of Carotid Diameter Stenosis **	**Before Radiotherapy **		**6 Months After Radiotherapy **		
		**Frequency**	**Percentage**	**Frequency**	**Percentage**	**P-value**
	Normal	32	94.1	15	44.1	
Right carotid	Less than 50%	2	5.9	19	55.9	>0.001*
	50-67	0	0	0	0	
	Above 70%	0	0	0	0	
	Normal	33	91.7	19	52.8	
Left carotid	Less than 50%	3	8.3	17	47.2	>0.001*
	50-67	0	0	0	0	
	Above 70%	0	0	0	0	
	Normal	65	92.9	34	48.6	
Sum of both	Less than 50%	5	7.1	36	51.4	>0.001*
	50-67	0	0	0	0	
	Above 70%	0	0	0	0	

McNemar Test^*^

In this research, 49 patients (mean age=59.46 years) were studied, of which 32 (65.3%) were male. Table-1 demonstrates the demographic characteristics of the investigated patients. As seen in Table-3, the results of the McNemar statistical test demonstrated that EDV did not show a statistically significant change at 6 months after radiotherapy. Table-4 shows that radiotherapy can cause significant carotid artery stenosis (P<0.001). Among 65 carotid arteries with normal diameters before radiotherapy, 31 (47.7%) developed less than 50% stenosis after radiotherapy. Table-5, shows that ICA/CCA PSV ratio did not change significantly during the study period. Table-6, demonstrates that the mean score of the middle intimal membrane thickness and the number of plaques of carotid arteries had a statistically significant difference 6 months after radiotherapy (P<0.05).

## Discussion

**Table:** Table5. Comparison of the Ratio of PSV of the Internal Carotid Artery to PSV of the Common Carotid Artery before and 6 Months after Radiotherapy

**Carotid Type**	**ICA/CCA PSV Ratio **	**Before Radiotherapy **		**6 Months After Radiotherapy **		
		**Frequency**	**Percentage**	**Frequency**	**Percentage**	**P-value**
	Less than 2	32	94.1	30	88.2	
Right carotid	2-4	2	5.9	4	11.8	0.500*
	More than 4	0	0	0	0	
	Less than 2	33	91.7	30	83.3	
Left carotid	2-4	3	8.3	6	16.7	0.250*
	More than 4	0	0	0	0	
	Less than 2	65	92.9	60	85.7	
Sum of both	2-4	5	7.1	10	14.3	0.063*
	More than 4	0	0	0	0	

**ICA/CCA PSV:** Internal carotid artery/ common carotid artery peak systolic velocity, McNemar Test^*^

**Table:** Table6. Comparison of the Middle Intimal Membrane Thickness and the Number of Plaques in Carotid Arteries before and 6 Months after Radiotherapy

**Variable**	**Carotid Type**	**Before Radiotherapy **		**6 Months After Radiotherapy **		
		**Frequency**	**Percentage**	**Frequency**	**Percentage**	**P-value**
	Right carotid	0.67	0.22	0.77	0.12	0.027*
Middle intimal membrane thickness	Left carotid	0.81	0.14	0.88	0.08	0.017*
	Sum of both	0.74	0.12	0.83	0.20	0.001*
	Right carotid	1.08	0.71	1.94	1.09	>0.001*
Number of vascular plaques	Left carotid	1.02	0.60	2.13	1.04	>0.001*
	Sum of both	1.05	0.65	2.04	1.06	>0.001*

PairedT-test^*^

The impact of neck radiotherapy on the results of carotid artery Doppler ultrasound in patients with HNC is the subject of ongoing research. Radiotherapy is the main treatment modality for many head and neck malignancies, and many patients survive long enough to experience its complications such as carotid artery damage [21]. Results of this study, which examined the effects of radiotherapy on neck vessels, revealed that radiotherapy does not have a short-term impact on PSV, EDV, and ICA/CCA ratio, whereas it increases the rate of carotid artery stenosis, middle intimal membrane thickness, and the number of vascular plaques within 6 months following its completion.

Even prophylactic reduced-dose radiotherapy to the neck lymph nodes (45-46 Gy, 1.8-2 Gy per fraction) led to increased middle intimal membrane thickness and increased number of plaques, according to the results. Our results are similar to that of Carmody et al.’s study, suggesting that high-dose radiotherapy might be an important risk factor for accelerating carotid atherosclerosis [25]. In line with our research, Lam et al. in their study showed that patients with nasopharyngeal carcinoma are prone to develop radiation-induced carotid artery stenosis. As a result, regular follow-up ultrasound examinations seemed to be essential for early diagnosis and potential therapeutic interventions in this group of patients [26].

Elerding et al. by investigating the incidence of carotid artery disorders after external neck radiotherapy of head and neck cancer recommended that all survivors undergo non-invasive vascular studies in their follow-up visits, especially five years after radiotherapy had finished [27].

Dubec et al. in their study on 45 patients stated that radiotherapy adversely impacted large arteries and routine color Doppler examinations at follow-ups should be considered for patients receiving head and neck radiotherapy [28]. Results of another study evaluating carotid artery stenosis after radiation therapy for nasopharyngeal carcinoma demonstrated that patients had an increased risk of developing carotid artery stenosis approximately five years after completion of radiotherapy [29]. In their study investigating the impacts of radiotherapy on carotid arteries in patients with HNC, Akhavan et al. reported that radiotherapy increased the number of plaques in the arteries but had no significant effect on the middle intimal membrane thickness [21].

Other studies have indicated that radiotherapy can result in increased intima-media thickness and subsequently carotid artery stenosis, which increases the risk of cerebrovascular accidents, such as transient ischemic attack and stroke [30][31].

The exact pathophysiology of radiation-induced carotid artery stenosis is still unknown. One important mechanism might be the dysfunction of endothelial cells that are highly sensitive to radiation. Damaged endothelial cells fail to function as a barrier against plasma lipoproteins. As a result, lipid filtration can activate the lysosomal system and result in the proliferation of endothelial cells [30].

In addition, radiation-induced atherosclerosis can cause carotid artery stenosis. Clinical evaluation of radiation-induced carotid injury includes diagnosis of newly formed lesions, adjustment of radiation and injury site, and consideration of the time between radiation and onset of symptoms [32]. It can be hypothesized that radiation-induced carotid stenosis is a potentially fatal complication of head and neck radiotherapy, and that its diagnosis and treatment could be lifesaving for cancer survivors.

In this study, PSV, EDV, and ICA/CCA ratio remained stable in the patients. This is similar to the results of Akhavan et al. study that concluded that radiation therapy probably has minimal effects on carotid blood flow parameters [21]. In contrast to the results of our study, the results of the study by Mohammad Karim et al showed that the hemodynamic parameters of CCA change as a result of radiotherapy, and these changes can lead to late complications such as ischemic stroke [33].

A Comparison of the results of this study with previous studies indicates that the hemodynamic parameters of the common carotid arteries can change during radiotherapy and cause late complications. Meticulous attention to carotid arteries in radiotherapy planning and avoidance of hot spots can -to some extent- alleviate the impacts of radiation on carotid arteries. This sequela should not be considered benign and should be treated in the same way as other causes of carotid stenosis.

## Conclusion

Carotid artery stenosis seems to be a common vascular complication of neck irradiation, and its long-term consequences may take years to manifest clinically. Radiotherapy probably has minimal effects on other ultrasound parameters of carotid arteries including EDV, PSV, and ICA/CCA ratio. A more accurate assessment of the studied parameters and the study of the clinical significance of these changes require a longer follow-up.

## Acknowledgment

This thesis has been extracted from the research project of the assistantship thesis approved by the Research Vice-Chancellor of the Faculty of Medicine at Shahid Beheshti University of Medical Sciences and Healthcare Services, Tehran (code of ethics: IR.SBMU.MSP.REC.1401.263). We would like to thank all the esteemed authorities of Imam Hossein Hospital, the esteemed staff of the Oncology and Radiology wards, and all the esteemed staff and the Research Vice-Chancellor of the Faculty of Medicine at Shahid Beheshti University of Medical Sciences and Healthcare Services, Tehran, for their sincere cooperation.

## Conflict of Interest

None declared.

## References

[R1] Da Cunha, Compton K, Xu R, Mishra R, Drangsholt MT, Antunes JLF, et al (2023). The global, regional, and national burden of adult lip, oral, and
pharyngeal cancer in 204 countries and territories: A systematic analysis for the
global burden of disease study 2019. JAMA Oncol.

[R2] Bhat GR, Hyole RG, Li J (2021). Head and neck cancer: Current challenges and future perspectives. Adv Cancer Res.

[R3] Mody MD, Rocco JW, Yom SS, Haddad RI, Saba NF (2021). Head and neck cancer. Lancet.

[R4] King SN, Dunlap NE, Tennant PA, Pitts T (2016). Pathophysiology of Radiation-Induced Dysphagia in Head and Neck Cancer. Dysphagia.

[R5] Rettig EM, D'Souza G (2015). Epidemiology of head and neck cancer. Surg Oncol Clin N Am.

[R6] Barati Sedeh, Poursheykhi H, Rezaei F (2019). Study of Thermal Effects of Laser on the Treatment of Head and Neck
Cancer with Multi-Layered Nanoparticles. J Lasers Med.

[R7] Vissink A, Jansma J, Spijkervet FK, Burlage FR, Coppes RP (2003). Oral sequelae of head and neck radiotherapy. Crit Rev Oral Biol Med.

[R8] Wu TT, Zhou SH (2015). Nanoparticle-based targeted therapeutics in head-and-neck cancer. Int J Med Sci.

[R9] Ferlito A, Shaha AR, Silver CE, Rinaldo A, Mondin V (2001). Incidence and sites of distant metastases from head and neck cancer. ORL J Otorhinolaryngol Relat Spec.

[R10] Schantz SP, Yu GP (2002). Head and neck cancer incidence trends in young Americans, 1973-1997, with
a special analysis for tongue cancer. Arch Otolaryngol Head Neck Surg.

[R11] Sakagami H (2010). Apoptosis-inducing activity and tumor-specificity of antitumor agents
against oral squamous cell carcinoma. Jpn Dent Sci Rev.

[R12] Adelstein DJ, Li Y, Adams GL, Wagner H, Kish JA, Ensley JF, Schuller DE, Forastiere AA (2003). An intergroup phase III comparison of standard radiation therapy and two
schedules of concurrent chemoradiotherapy in patients with unresectable squamous
cell head and neck cancer. J Clin Oncol.

[R13] Pignon JP, le Maître, Maillard E, Bourhis J; (2009). Meta-analysis of chemotherapy in head and neck cancer (MACH-NC): an
update on 93 randomised trials and 17,346 patients. Radiother Oncol.

[R14] Vermorken JB, Mesia R, Rivera F, Remenar E, Kawecki A, Rottey S, Erfan J, Zabolotnyy D, Kienzer HR, Cupissol D, Peyrade F, Benasso M, Vynnychenko I, De Raucourt, Bokemeyer C, Schueler A, Amellal N, Hitt R (2008). Platinum-based chemotherapy plus cetuximab in head and neck cancer. N Engl J Med.

[R15] Mellière D, Becquemin JP, Hoehne M, Dermer J, Carlier R (1983). Vraies et fausses artérites radiques [True and false radiation
arteritis]. J Mal Vasc.

[R16] Semba SE, Mealey BL, Hallmon WW (1994). The head and neck radiotherapy patient: Part 1-Oral manifestations of
radiation therapy. Compendium.

[R17] Kırca K, Kutlutürkan S (2017). Symptoms of patients with head and neck cancers undergoing radiotherapy. Eur J Cancer Care (Engl).

[R18] Head MA, Kamal M, Rosenthal DI, Volpe S, Goepfert RP, Garden AS, Hutcheson KA, Al Feghali, Meheissen MA, Eraj SA, Dursteler AE (2018). Patient reported dry mouth: instrument comparison and model performance
for correlation with quality of life in head and neck cancer survivors. Radiotherapy and Oncology.

[R19] Lee AW, Ng SH, Ho JH, Tse VK, Poon YF, Tse CC, Au GK, O SK, Lau WH, Foo WW (1988). Clinical diagnosis of late temporal lobe necrosis following radiation
therapy for nasopharyngeal carcinoma. Cancer.

[R20] Lam KS, Tse VK, Wang C, Yeung RT, Ho JH (1991). Effects of cranial irradiation on hypothalamic-pituitary function--a
5-year longitudinal study in patients with nasopharyngeal carcinoma. Q J Med.

[R21] Akhavan A, Farghadani M, Emami H, Naderi Beni, Naderi Beni (2021). Effects of Neck Radiation on Result of Doppler Sonography of Carotid
Arteries in Head and Neck Cancer Patients. J Stroke Cerebrovasc Dis.

[R22] Weintraub NL, Jones WK, Manka D (2010). Understanding radiation-induced vascular disease. J Am Coll Cardiol.

[R23] Scoutt LM, Gunabushanam G (2019). Carotid Ultrasound. Radiol Clin North Am.

[R24] Lee W (2014). General principles of carotid Doppler ultrasonography. Ultrasonography.

[R25] Carmody BJ, Arora S, Avena R, Curry KM, Simpkins J, Cosby K, Sidawy AN (1999). Accelerated carotid artery disease after high-dose head and neck
radiotherapy: is there a role for routine carotid duplex surveillance. J Vasc Surg.

[R26] Lam WW, Leung SF, So NM, Wong KS, Liu KH, Ku PK, Yuen HY, Metreweli C (2001). Incidence of carotid stenosis in nasopharyngeal carcinoma patients after
radiotherapy. Cancer.

[R27] Elerding SC, Fernandez RN, Grotta JC, Lindberg RD, Causay LC, McMurtrey MJ (1981). Carotid artery disease following external cervical irradiation. Ann Surg.

[R28] Dubec JJ, Munk PL, Tsang V, Lee MJ, Janzen DL, Buckley J, Seal M, Taylor D (1998). Carotid artery stenosis in patients who have undergone radiation therapy
for head and neck malignancy. Br J Radiol.

[R29] Cheng SW, Ting AC, Lam LK, Wei WI (2000). Carotid stenosis after radiotherapy for nasopharyngeal carcinoma. Arch Otolaryngol Head Neck Surg.

[R30] Xu J, Cao Y (2014). Radiation-induced carotid artery stenosis: a comprehensive review of the
literature. Interv Neurol.

[R31] Trojanowski P, Sojka M, Trojanowska A, Wolski A, Roman T, Jargiello T (2019). Management of Radiation Induced Carotid Stenosis in Head and Neck
Cancer. Transl Oncol.

[R32] Mullen E (2023). Radiation-Induced Carotid Artery Stenosis: What Nurses Need to Know. Clin J Oncol Nurs.

[R33] Mohammadkarim A, Mokhtari-Dizaji M, Kazemian A, Saberi H (2018). Hemodynamic analysis of radiation-induced damage in common carotid
arteries by using color Doppler ultrasonography. Ultrasonography.

